# Platelet Proteomics and Tissue Metabolomics Investigation for the Mechanism of Aspirin Eugenol Ester on Preventive Thrombosis Mechanism in a Rat Thrombosis Model

**DOI:** 10.3390/ijms251910747

**Published:** 2024-10-06

**Authors:** Qi Tao, Li-Ping Fan, Ji Feng, Zhi-Jie Zhang, Xi-Wang Liu, Zhe Qin, Jian-Yong Li, Ya-Jun Yang

**Affiliations:** Key Lab of New Animal Drug of Gansu Province, Key Lab of Veterinary Pharmaceutical Development of Ministry of Agriculture and Rural Affairs, Lanzhou Institute of Husbandry and Pharmaceutical Sciences of CAAS, Lanzhou 730050, China; taoqi19951224@163.com (Q.T.); fanliping43t@163.com (L.-P.F.); fengji17520511105@163.com (J.F.); 18793107830@163.com (Z.-J.Z.); xiwangliu@126.com (X.-W.L.); qinzhe@caas.cn (Z.Q.)

**Keywords:** aspirin eugenol ester (AEE), thrombosis, platelet, proteomics, metabolomics, molecular docking

## Abstract

Platelet activation is closely related to thrombosis. Aspirin eugenol ester (AEE) is a novel medicinal compound synthesized by esterifying aspirin with eugenol using the pro-drug principle. Pharmacological and pharmacodynamic experiments showed that AEE has excellent anti-inflammatory, antioxidant, and inhibitory platelet activation effects, preventing thrombosis. However, the regulatory network and action target of AEE in inhibiting platelet activation remain unknown. This study aimed to investigate the effects of AEE on platelets of thrombosed rats to reveal its regulatory mechanism via a multi-omics approach. The platelet proteomic results showed that 348 DEPs were identified in the AEE group compared with the model group, of which 87 were up- and 261 down-regulated. The pathways in this result were different from previous results, including mTOR signaling and ADP signaling at P2Y purinoceptor 12. The metabolomics of heart and abdominal aortic tissue results showed that the differential metabolites were mainly involved in steroid biosynthesis, the citric acid cycle, phenylalanine metabolism, phenylalanine, tyrosine, and tryptophan biosynthesis, and glutathione metabolism. Molecular docking results showed that AEE had a better binding force to both the COX-1 and P2Y12 protein. AEE could effectively inhibit platelet activation by inhibiting COX-1 protein and P2Y12 protein activity, thereby inhibiting platelet aggregation. Therefore, AEE can have a positive effect on inhibiting platelet activation.

## 1. Introduction

A thrombus is a solid mass formed in the heart and blood vessels of the organism when blood coagulates or certain formed components of the blood coagulate, which can lead to myocardial infarction, ischemic stroke, and other cardiovascular diseases (CVD) [1]. From the 1990s to the present day, the number of CVD patients has been increasing year by year. CVD has become a major health challenge for the population and a leading cause of death [2,3]. Thrombus formation is a complex process that involves various cells and regulatory factors and has many causes, but one of the main reasons for its formation is platelet activation [4]. Platelets originate from segmented breaks in the cytoplasm of megakaryocytes. Platelet morphology shows a smooth surface, slightly raised on both sides of the disc, with an average diameter of 2~4 μm; its surface structure consists of the cell coat and the cell membrane. The cell coat is mainly composed of glycoproteins and glycoproteins glycan chains, and the platelet cell membrane contains a variety of enzymes and coagulation-related receptors, such as protease-activated receptor, adenosine diphosphate (ADP) receptor, thromboxane A2(TXA2) receptor, and glycoprotein receptor GPIb/IX/V complexes [5,6,7]. Platelets have a variety of granule components, including α granules, dense granules, and lysosomes, which contain several coagulation-related factors. Platelets lack spontaneous aggregation in normal circulation. When blood vessels are damaged, platelets adhere to the surface of the damaged vessel and release bioactive substances that enable the recruitment of additional platelets from the circulation. Subsequently, the platelet production of TXA2 and ADP triggers platelet aggregation [8,9].

With the development of medicine and an increasing understanding of thrombotic diseases, there has been great progress in developing novel antithrombotic drugs. However, all have varying degrees of bleeding risk [10,11]. For example, the long-term use of COX-1 antagonist aspirin can result in the long-term inhibition of COX-1, which leads to insufficient PG synthesis and reduced mucosal resistance, thereby leading to gastrointestinal bleeding [12]. P2Y12 antagonists (ticagrelor and cangrelor) were used to treat acute coronary syndromes but had a certain probability of causing adverse effects such as neutropenia and cerebral hemorrhage [13]. Tirofiban, an integrin αIIbβ3 antagonist, is used clinically in the treatment of acute ischaemic stroke, angina pectoris, and myocardial infarction. However, there is a certain probability of causing local bleeding and thrombocytopenia in the organism [14,15]. TB-402 was a long-acting anti-FVIII monoclonal antibody that underwent clinical phase I and II trials. Unfortunately, TB-402 was associated with significantly higher incidents of hemorrhage than rivaroxaban in both the high- and low-dose groups, the drug development of which has been terminated [16]. TTP889 was the first small molecule inhibitor of FIXa with oral administration. However, the dosage of TTP889 300 mg was not effective in the prevention of venous thromboembolism in this setting, and the development of TTP889 was eventually stopped [17]. Therefore, it is increasingly important to develop novel thromboprophylaxis drugs that are more effective and safer.

Aspirin eugenol ester (AEE) is a novel medicinal compound synthesized by the esterification of aspirin with eugenol using the pro-drug principle. Eugenol has various pharmacological activities such as anti-inflammatory, antipyretic, antithrombotic, antioxidant, and analgesic effects, but the instability and pungent odor exhibited by eugenol limited its application [18,19,20]. AEE maintains the pharmacological activity of aspirin and eugenol, masks the carboxyl group of aspirin, reduces the side effects of aspirin on the gastrointestinal tract when it enters the body, and overcomes the pungent odor and instability of eugenol. H_2_O_2_ was applied to HUVECs (Human umbilical vein endothelial cells) after dividing them into AEE-pretreated and untreated groups, respectively, and the AEE pretreatment of HUVECs increased the glutathione/oxidized glutathione ratio and activities of superoxide dismutase and glutathione peroxidase and reduced the damage to the antioxidant system caused by H_2_O_2_ [21]. NO has antithrombotic and vasodilatory effects. In healthy blood vessels, the intact endothelium releases NO to inhibit platelet adhesion and aggregation on the endothelium [22,23]. Further research showed that H_2_O_2_-induced dysregulation of the NO regulatory system in HUVECs was significantly ameliorated by AEE treatment. The related adhesion factors on oxidatively damaged vascular endothelial cells were significantly changed, in which the expression of E-selectin increased considerably on damaged vascular endothelial cells, and the expression of E-selectin was significantly down-regulated on damaged vascular endothelial cells after AEE treatment in both in vivo and in vitro models of vascular endothelial oxidative injury. AEE was effective at reducing the adhesion of oxidatively damaged HUVECs to macrophages (THP-1) [24]. This indicated that the thromboprophylaxis of AEE may be undertaken by protecting vascular endothelial cells from the damage caused by external stimuli and reducing the expression of adhesion factors and adhesion to macrophages (THP-1). It was investigated for the effect of AEE on five proteins associated with inflammation and thrombosis, including COX-1, COX-2, C reactive protein, prothrombin (FII), and arachidonic acid 5-lipoxygenase. The results showed that in the comparison of the strength of the pharmacological effects on the five proteins, the AEE group was stronger than the aspirin and eugenol groups [25]. AEE inhibits platelet aggregation and intracellular calcium concentration by inhibiting TXA2 production and finally inhibits extracellular signal-regulated kinase (ERK2) activity, followed by the inhibition of the AMPK/Sirt 1 pathway, leading to reduced CD40L expression, thereby inhibiting ATP release and the PI3K/Akt pathway [26,27].

With the rapid development of biotechnology, omics research has made remarkable progress in recent years [28]. Omics technology provides us with a comprehensive and systematic means of understanding biological systems, which can provide comprehensive biological information, reveal the complexity and dynamics of biological systems, promote interdisciplinary research, and accelerate drug discovery and development. It is of great significance for solving practical problems.

Multi-omics studies in the plasma and liver showed that AEE could regulate platelet activation and other pathways to prevent thrombosis [29,30]. This indicates that AEE has a definite thromboprophylaxis- and platelet activation-inhibiting effect. However, the molecular mechanism and target of AEE to inhibit platelet activation remains unknown. Therefore, this study used platelet proteomics and tissue metabolomics approaches to explore the regulatory network and action target of AEE to inhibit platelet activation, which provides theoretical support and ideas for developing AEE into an antiplatelet and thrombopreventive drug.

## 2. Results

### 2.1. Identification and Quantitative Evaluation of Proteins

Four-dimensional proteomics adds a fourth dimension, ion mobility, to the three dimensions of three-dimensional separation, i.e., retention time, mass-to-charge ratio (*m*/*z*), and ion intensity, dramatically improving scanning speed and detection sensitivity. Four-dimensional label-free proteomic analysis of rat platelets showed that 2315, 1783, and 1545 proteins were identified in the control, model, and AEE groups, respectively (Figure S1A). A total of 3134 proteins and 28,322 peptides were identified (Figure S1B). As shown in Figure S1C–E, 98.6% of the proteins had a mass of ≥10 kDa, and >89.8% of the proteins contained at least two peptides, with most of the peptides distributed over 7~20 amino acids, thus indicating that the data were of good quality. Moreover, 79.3% of the peptide sequences had a coverage distribution >10 (Figure S1F), which indicated the high quality of the proteomic data.

### 2.2. DEPs in Rat Platelet

**Figure 1:**
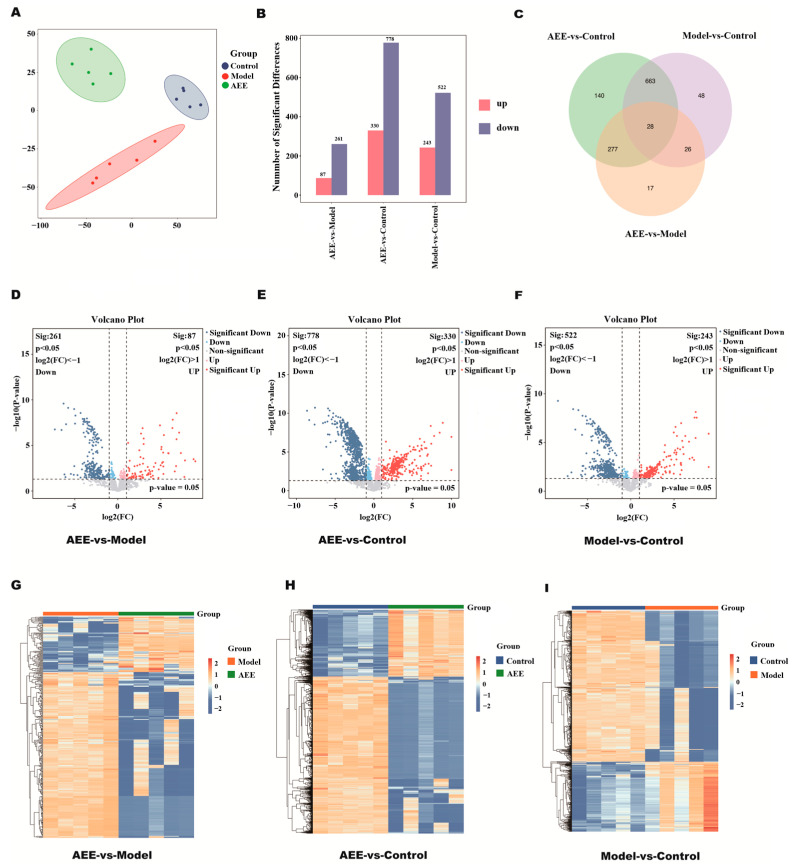
Changes in rat platelets. (**A**) Principal component analysis of normalized protein expression. (**B**) Histogram of DEP statistics. (**C**) Venn diagram of DEPs. (**D**) Volcano plot of DEPs between AEE and model groups. (**E**) Volcano plot of DEPs between AEE and control groups. (**F**) Volcano plot of DEPs between model and control groups. (**G**) Heat map of DEPs between AEE and model groups. (**H**) Heat map of DEPs between AEE and control groups. (**I**) Heat map of DEPs between model and control groups.

To evaluate between-group differences and within-group sample duplications, PCA was performed on the readings of all samples in this study, which showed small differences within each group of platelet samples and large differences in the distribution between the three groups (Figure 1A). As shown in Figure 1B–I, 348 DEPs were identified in the AEE and model groups. In total, 87 up-regulated and 261 down-regulated proteins were identified in the AEE group compared to the model group. A total of 1108 DEPs were identified between the AEE group and the control group, of which 330 proteins were up-regulated while 778 proteins were down-regulated. A total of 765 DEPs were found between the model and control groups, of which 243 were up-regulated while 522 were down-regulated proteins. The Venn diagram shows the overlap of DEPs, with a total of 28 DEPs in the three groups. A total of 140, 17, and 48 DEPs were specifically changed in the AEE vs. control, AEE vs. model, and model vs. control, respectively. The expression patterns of the DEPs in each sample were clustered according to a *p*-value less than 0.05 and the 2-fold change values (or lower than 0.5) of their expression ratios, and these results showed good reproducibility of the samples, with significant changes in protein expression in each group. These indicated that platelet protein expression could be significantly changed in thrombosed rats after AEE treatment.

### 2.3. Reactome Pathway Enrichment of DEPs

**Figure 2:**
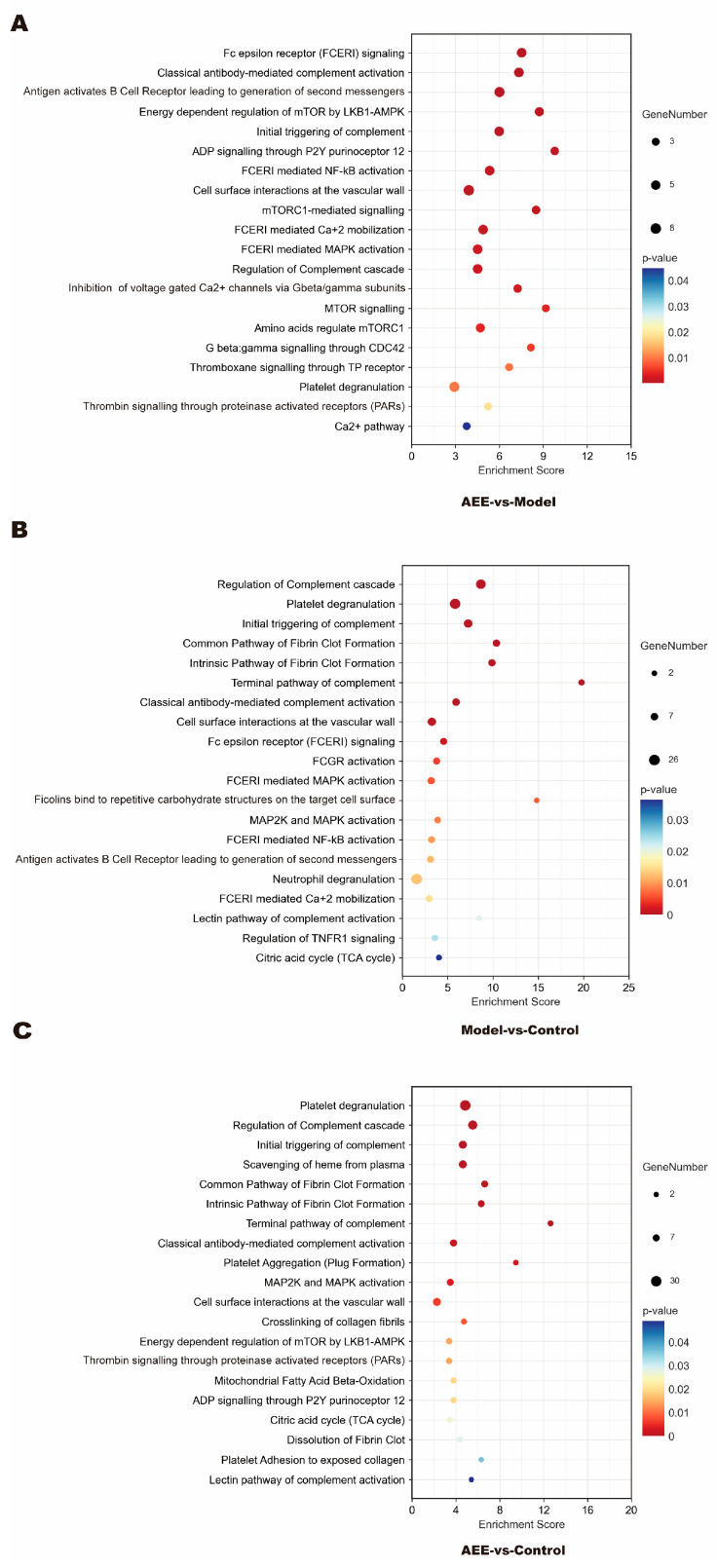
Reactome pathway analysis of DEPs. (**A**) AEE group vs. model group. (**B**) Model group vs. control group. (**C**) AEE group vs. control group.

Reactome is a free, open-source relational database of signaling and metabolic molecules organized into biological pathways and processes (https://reactome.org/, accessed 16 April 2024). In this study, the obtained DEPs were imported into the Reactome pathway database and analyzed. Reactome analysis showed that between the AEE and model groups, DEPs were mainly enriched in fc epsilon receptor signaling, classical antibody-mediated complement activation, and the antigen-activated B cell receptor, leading to the generation of second messengers, the initial triggering of a complement, ADP signaling through P2Y purinoceptor 12, FCERI-mediated NF-κB activation, cell surface interactions at the vascular wall, the regulation of complement cascade, mTOR signaling, thromboxane signaling through the TP receptor, platelet degranulation, thrombin signaling through proteinase-activated receptors, and the Ca^2+^ pathway (Figure 2A). Between the model and control groups, DEPs were mainly enriched in the regulation of complement cascade, platelet degranulation, the initial triggering of complement, the common pathway of fibrin clot formation, the intrinsic pathway of fibrin clot formation, the terminal pathway of complement, classical antibody-mediated complement activation, cell surface interactions at the vascular wall, FCERI-mediated NF-κB activation, FCERI-mediated MAPK activation, neutrophil degranulation, the lectin pathway of complement activation, and the citric acid cycle (Figure 2B). As shown in Figure 2C, between the AEE and control groups, DEPs were mainly enriched in platelet degranulation, the regulation of complement cascade, the initial triggering of complement, the scavenging of heme from plasma, the common pathway of fibrin clot formation, the intrinsic pathway of fibrin clot formation, the terminal pathway of complement, classical antibody-mediated complement activation, platelet aggregation, MAP2K and MAPK activation, cell surface interactions at the vascular wall, the cross-linking of collagen fibrils, thrombin signaling through proteinase-activated receptors, ADP signaling through P2Y purinoceptor 12, the citric acid cycle, the dissolution of fibrin clot, platelet adhesion to exposed collagen, and the lectin pathway of complement activation.

### 2.4. GO Enrichment of DEPs

**Figure 3:**
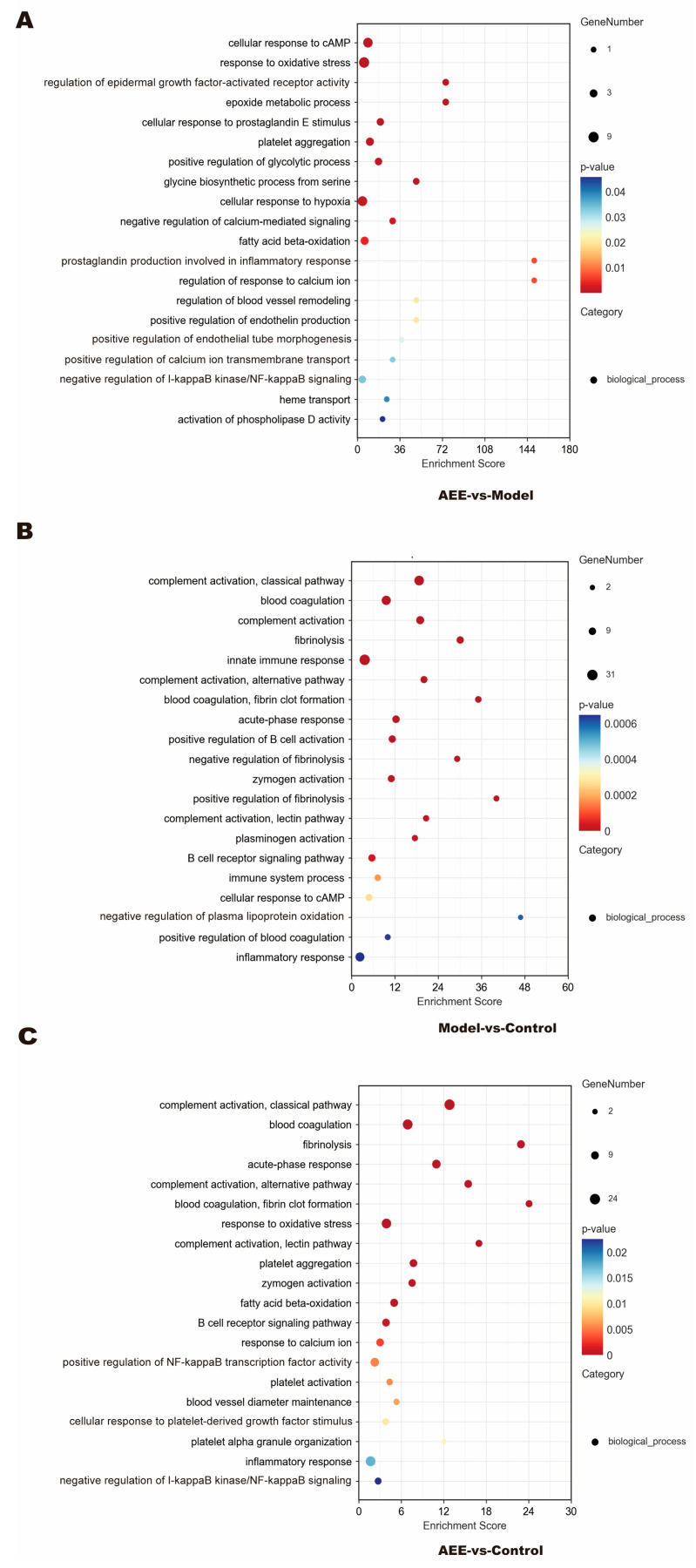
GO enrichment analysis of DEPs. (**A**) AEE group vs. model group. (**B**) Model group vs. control group. (**C**) AEE group vs. control group.

GO is a database created by the Gene Ontology Consortium to establish a standard for a semantic vocabulary that applies to a wide range of species, qualifies and describes the function of genes and proteins, and can be updated as research progresses. To better understand the biological functions of DEPs, the obtained DEPs were imported into the GO database and analyzed in this study, which mainly explored the functions of DEPs in the biological process (BP) category. Between the AEE and model groups, DEPs were mainly enriched in cellular response to cAMP, in response to oxidative stress, the regulation of epidermal growth factor-activated receptor activity, epoxide metabolic processes, cellular response to prostaglandin E stimulus, platelet aggregation, the positive regulation of glycolytic process, fatty acid beta-oxidation, prostaglandin production involved in the inflammatory response, the regulation of response to calcium ions, the regulation of blood vessel remodeling, the positive regulation of endothelin production, the positive regulation of endothelial tube morphogenesis, the positive regulation of calcium ion transmembrane transport, the negative regulation of I-κB kinase/NF-κB signaling, and heme transport (Figure 3A). Most of these DEPs were adjusted downward (Figure S2A). Between the model and control groups, DEPs were mainly enriched in complement activation, blood coagulation, fibrinolysis, innate immune response, acute-phase response, the positive regulation of B cell activation, zymogen activation, plasminogen activation, immune system processes, the cellular response to cAMP, and inflammatory response (Figure 3B). Most of these DEPs were adjusted upward (Figure S2B). As shown in Figure 3C and Figure S2C, between the AEE and control groups, DEPs were mainly enriched in complement activation, blood coagulation, fibrinolysis, acute-phase response, response to oxidative stress, platelet aggregation, zymogen activation, fatty acid beta-oxidation, the B cell receptor signaling pathway, the positive regulation of NF-κB transcription factor activity, platelet activation, blood vessel diameter maintenance, response to calcium ions, cellular response to platelet-derived growth factor stimulus, platelet alpha granule organization, inflammatory response, and the negative regulation of I-κB kinase/NF-κB signaling.

### 2.5. KEGG Pathway Enrichment of DEPs

**Figure 4:**
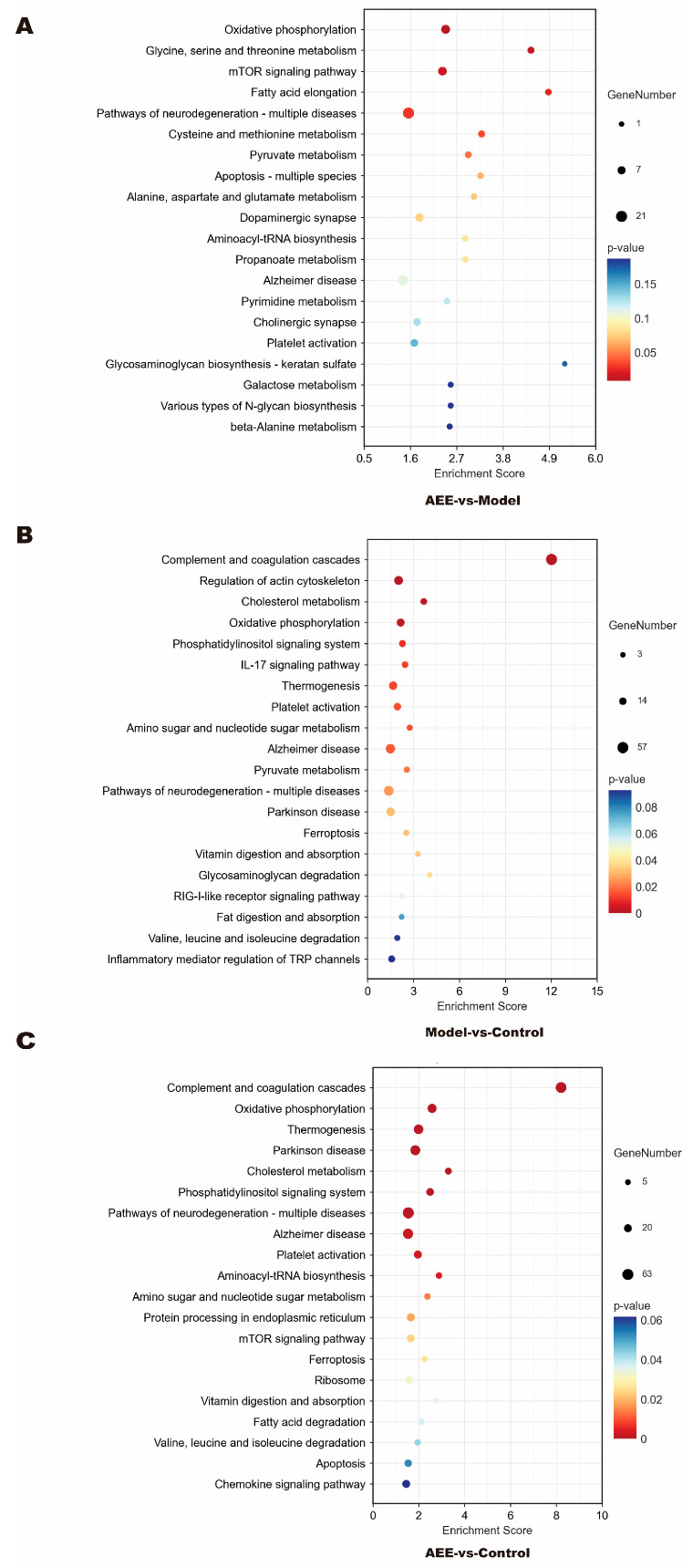
KEGG pathway analysis of DEPs. (**A**) AEE group vs. model group. (**B**) Model group vs. control group. (**C**) AEE group vs. control group.

The KEGG database was launched in 1995 by Kanehisa Laboratories as an online database of genomes, enzymatic pathways, and biochemicals. To identify the pathways in which DEPs were involved, the DEPs obtained in this study were imported into the KEGG pathway database for analysis. Between the AEE and model groups, DEPs were mainly enriched in oxidative phosphorylation, glycine, serine and threonine metabolism, the mTOR signaling pathway, cysteine and methionine metabolism, pyruvate metabolism, apoptosis—multiple species, alanine, aspartate and glutamate metabolism, platelet activation, galactose metabolism, and beta-alanine metabolism (Figure 4A and Figure S3A). Between the model and control groups, DEPs were mainly enriched in complement and coagulation cascades, the regulation of actin cytoskeleton, oxidative phosphorylation, the phosphatidylinositol signaling system, IL-17 signaling pathway, thermogenesis, platelet activation, amino sugar and nucleotide sugar metabolism, ferroptosis, valine, and leucine and isoleucine degradation (Figure 4B and Figure S3B). Between the AEE and control groups, DEPs were mainly enriched in complement and coagulation cascades, oxidative phosphorylation, thermogenesis, the phosphatidylinositol signaling system, platelet activation, aminoacyl-tRNA biosynthesis, amino sugar and nucleotide sugar metabolism, the mTOR signaling pathway, fatty acid degradation, valine, and leucine and isoleucine degradation (Figure 4C and Figure S3C). As shown in Figure S4A–C, in the platelet activation pathway map, compared with the model group, P2Y12, PLCβ, IP3R, Talin, and PI3K proteins were significantly down-regulated in the AEE group (*p* < 0.05). Compared with the control group, F2, FG proteins were significantly up-regulated, and PI3K, ERK proteins were significantly down-regulated in the model group (*p* < 0.05). Compared with the control group, P2Y12, PLCβ, IP3R, Talin, PI3K, and ERK proteins were significantly down-regulated, and F2, actin, and FG proteins were significantly up-regulated in the AEE group (*p* < 0.05).

### 2.6. PPI Analysis

**Figure 5:**
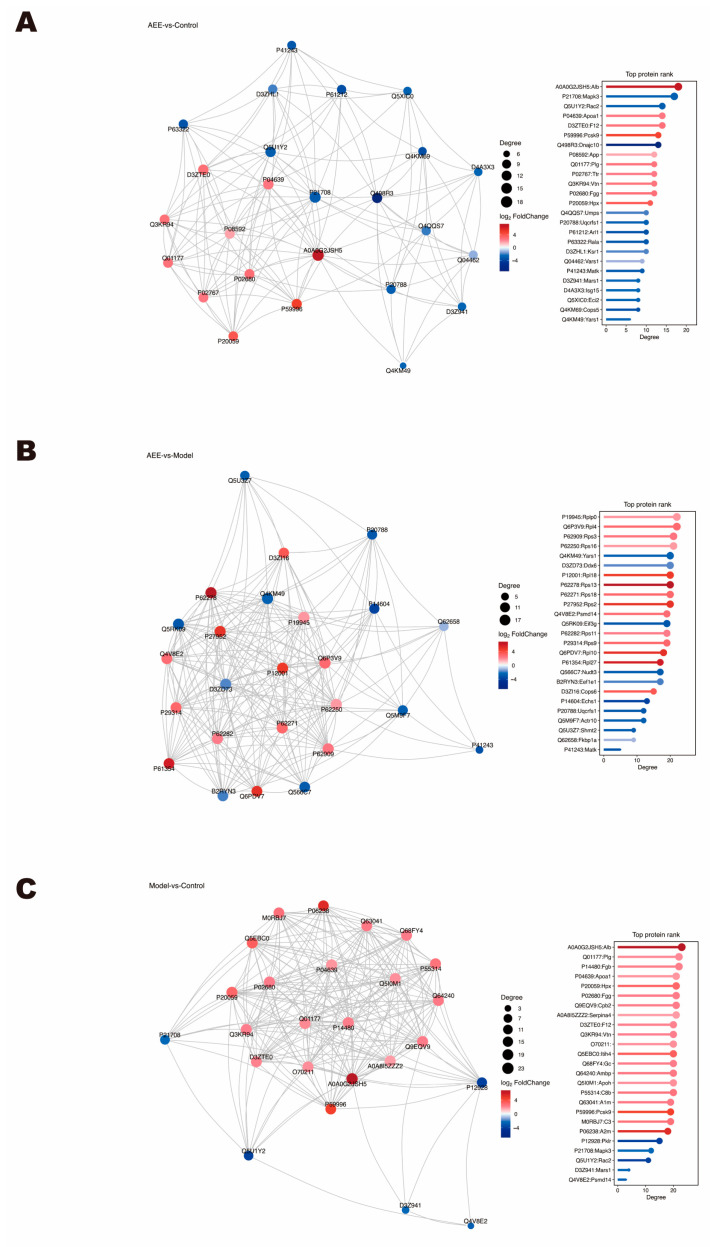
Interaction networks of the DEPs were analyzed using the STRING Database. (**A**) AEE group vs. control group. (**B**) AEE group vs. model group. (**C**) Model group vs. control group.

The interaction networks of the DEPs were analyzed using the STRING Database. Figure 5 shows that the PPI results were consistent with Reactome, GO, and KEGG pathway analysis. Between the AEE and control groups, these DEPs were associated with the positive regulation of fibrinolysis, the positive regulation of blood coagulation, platelet activation, platelet aggregation, complement and coagulation cascades, responses to stimulus, and inflammatory responses (Figure 5A). As for the DEPs in the AEE and model group, the PPI network (Figure 5B) indicated that some DEPs had a connection with response to elevated platelet cytosolic Ca^2+^, the platelet alpha granule, ADP signaling through P2Y purinoceptor 12, platelet degranulation, platelet activation, hemostasis, innate immune response in mucosa, and cellular response to cAMP. Between the model and control groups, these DEPs were associated with fibrinolysis, blood coagulation, fibrin clot formation, cell–matrix adhesion, plasminogen activation, complement activation, zymogen activation, acute inflammatory response, platelet aggregation, the activation of immune response, inflammatory response, responses to oxidative stress, platelet activation, and innate immune response (Figure 5C).

### 2.7. Metabonomics Analysis Results

#### 2.7.1. Analysis of Heart and Abdominal Aorta Metabolites

**Figure 6:**
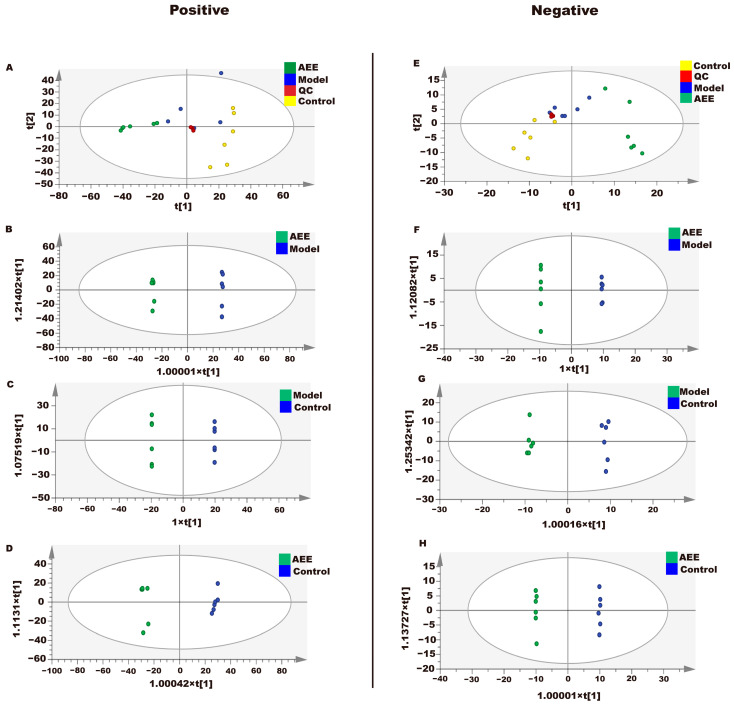
Effect of AEE on metabolomic profiles of hearts in thrombosed rats. The OPLS-DA score plots of different groups in ESI+ and ESI- modes. ESI+: electrospray ionization in positive ion mode; ESI-: electrospray ionization in negative ion mode. (**A**,**E**) The PCA score plots of the heart samples analyzed by the UPLC-Q-TOF/MS in ESI+ and ESI- modes. (**B**,**F**) The OPLS-DA score plots for AEE and model groups in ESI+ and ESI- models: ESI+: R^2^X = 0.842, R^2^Y = 1, Q^2^ = 0.949; ESI-: R^2^X = 0.789, R^2^Y = 1, Q^2^ = 0.968. (**C**,**G**) OPLS-DA score plots for model and control groups in ESI+ and ESI- models: ESI+: R^2^X = 0.605, R^2^Y = 1, Q^2^ = 0.851; ESI-: R^2^X = 0.791, R^2^Y = 0.997, Q^2^ = 0.797. (**D**,**H**) OPLS-DA score plots for AEE and control groups in ESI+ and ESI- models: ESI+: R^2^X = 0.452, R^2^Y = 0.996, Q^2^ = 0.931; ESI-: R^2^X = 0.723, R^2^Y = 1, Q^2^ = 0.949.

The metabonomic data of heart tissues in rats using UPLC-Q-TOF/MS are provided in File S1. The metabolite data of heart samples were analyzed by principal component analysis (PCA). According to the PCA score plots (Figure 6A,E), QC samples in ESI+ and ESI- were tightly clustered, indicating the stability and reproducibility of this method. In addition, the three groups separated well, indicating that the metabolomic profiles of the heart samples differed between the control, model, and AEE groups. To further reduce the influence of irrelevant factors on the analysis results and improve the separation and identification of metabolites, three experimental groups were modeled and analyzed using orthogonal partial least squares discriminant analysis (OPLS-DA). The results showed that the heart samples from three experimental groups are separated in Figure 6. The potential differential metabolites were screened by VIP > 1 and *p* < 0.05, and the differential metabolites were analyzed by a target MS/MS scan to obtain the secondary characteristic ion fragments of the differential metabolites, which could be used for comparisons in relevant databases. This study explored the change in metabolites between the AEE and model groups. As shown in Table S2, eight differential metabolites were finally screened in the heart samples analyzed, including hydroquinone, FAPy-adenine, 1-methylnicotinamide, phosphorylcholine, lipoamide, phosphorylcholine, 7-dehydrocholesterol, and 2-phenylbutyric acid.

**Figure 7:**
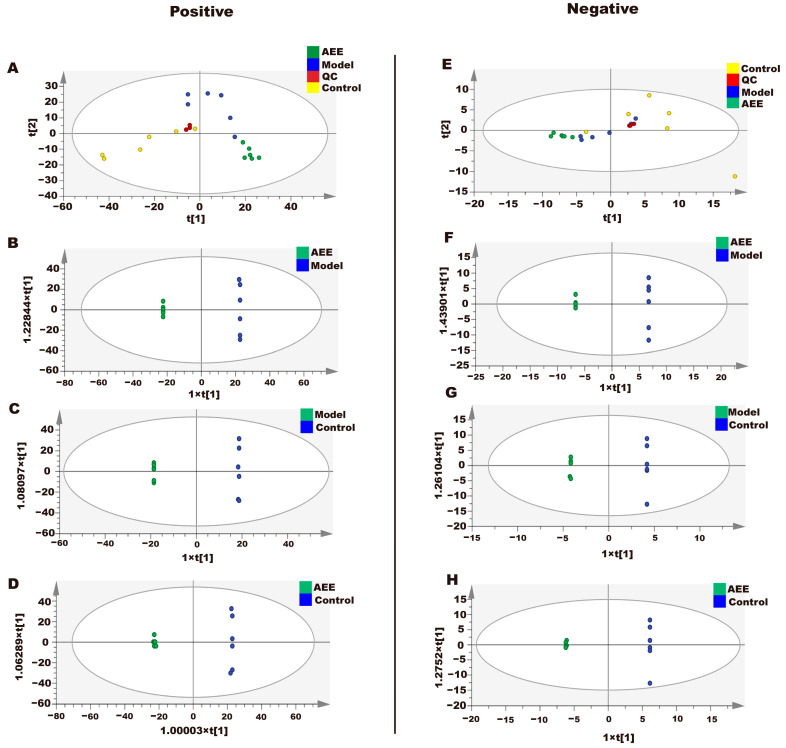
Effect of AEE on metabolomic profiles of abdominal aorta in thrombosed rats. The OPLS-DA score plots of different groups in ESI+ and ESI- modes. ESI+: electrospray ionization in positive ion mode; ESI-: electrospray ionization in negative ion mode. (**A**,**E**) PCA score plots of abdominal aorta samples analyzed by the UPLC-Q-TOF/MS in ESI+ and ESI- modes. (**B**,**F**) OPLS-DA score plots for AEE and model groups in ESI+ and ESI- models: ESI+: R^2^X = 0.750, R^2^Y = 1, Q^2^ = 0.973; ESI-: R^2^X = 0.889, R^2^Y = 1, Q^2^ = 0.953. (**C**,**G**) OPLS-DA score plots for model and control groups in ESI+ and ESI- models: ESI+: R^2^X = 0.765, R^2^Y = 1, Q^2^ = 0.944; ESI-: R^2^X = 0.883, R^2^Y = 0.1, Q^2^ = 0.875. (**D**,**H**) OPLS-DA score plots for AEE and control groups in ESI+ and ESI- models: ESI+: R^2^X = 0.681, R^2^Y = 1, Q^2^ = 0.959; ESI-: R^2^X = 0.871, R^2^Y = 1, Q^2^ = 0.986.

The metabonomic data of abdominal aorta tissue in rats using UPLC-Q-TOF/MS are provided in Supplementary File S2. The results showed that the abdominal aorta samples from three experimental groups were separated, as presented in Figure 7. The potential differential metabolites were screened by VIP > 1 and *p* < 0.05, to obtain the secondary characteristic ion fragments of the differential metabolites, which could be used for comparisons in relevant databases. This study explored the change in metabolites between the AEE and model groups. As shown in Table S3, ten differential metabolites were finally screened in the abdominal aorta samples analyzed, including glutathione, adenine, 1-methylnicotinamide, phenylacetaldehyde, desaminotyrosine, 2-methylglutaric acid, indolelactic acid, etiocholanolone, phenylalanine, and 3-phenylbutyric acid.

#### 2.7.2. Metabolic Pathway and Function Analysis

**Figure 8:**
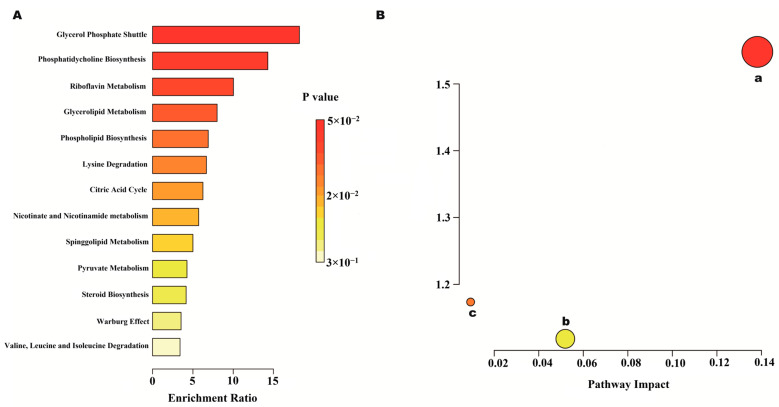
Heart metabolomics analysis results. (**A**) Metabolite enrichment analysis. (**B**) Metabolite pathway analysis. a: Nicotinate and nicotinamide metabolism; b: glycerophospholipid metabolism; and c: steroid biosynthesis.

**Figure 9:**
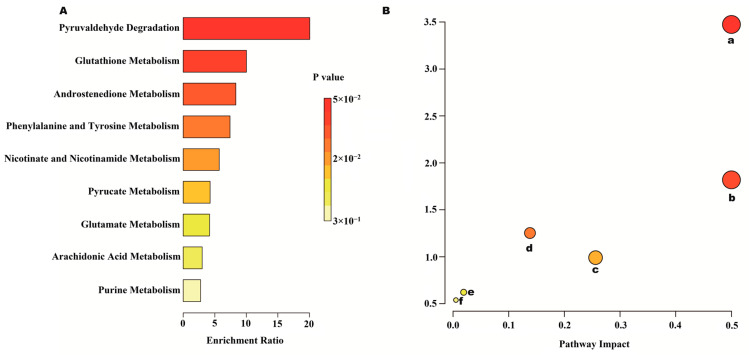
Abdominal aorta metabolomic analysis results. (**A**) Metabolite enrichment analysis. (**B**) Metabolite pathway analysis. a: Phenylalanine metabolism; b: phenylalanine, tyrosine, and tryptophan biosynthesis; c: nicotinate and nicotinamide metabolism; d: glutathione metabolism; e: purine metabolism; and f: steroid hormone biosynthesis.

To investigate the effects of AEE on metabolic pathways in the thrombosed rat organism, metabolic pathways were analyzed using MetaboAnalyst 6.0. Figure 8 shows an overview of the metabolic pathway analyses of heart metabolites in the form of bar and bubble plots. Main metabolic pathways include nicotinate and nicotinamide metabolism, glycerophospholipid metabolism, steroid biosynthesis, and the citric acid cycle. As shown in Figure 9, the metabolic pathways of abdominal aortic metabolites mainly include phenylalanine metabolism; phenylalanine, tyrosine, and tryptophan biosynthesis; nicotinate and nicotinamide metabolism; glutathione metabolism; purine metabolism; and steroid hormone biosynthesis.

### 2.8. Molecular Docking Studies

**Figure 10:**
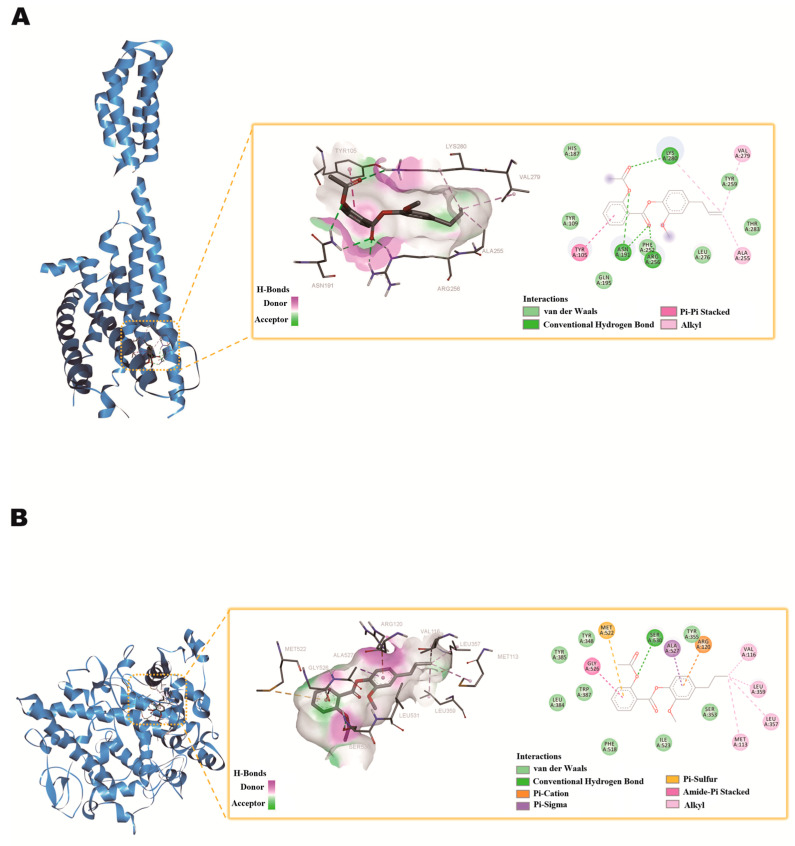
Docking pattern diagram. (**A**) AEE and protein P2Y12. (**B**) AEE and protein COX-1.

Based on the proteomics results, it was found that the expression of the P2Y12 protein in platelets of thrombosed rats was significantly decreased after treatment with AEE. Ticagrelor and cangrelor are both inhibitors of P2Y12. Aspirin can acetylate the hydroxyl group on the serine residue at position 530 of the COX-1 polypeptide chain, inactivating COX-1 and blocking TXA2 production, thereby inhibiting platelet activation. Therefore, this study performed molecular docking using AutoDock Vina software (version 1.2.0). The results showed that AEE, ticagrelor, and cangrelor could form binding sites with the P2Y12 protein as binding energies of −8.2, −9.2, and −6.9 kJ, respectively (Table S4). Six active site residues (LYS280, VAL279, ALA255, ARG256, ASN191, TYR105) were involved in AEE identification (Figure 10A). In protein P2Y12, the LYS280, ARG256, and ASN191 side chains formed conventional hydrogen bonds with AEE. The LYS280, VAL279, and ALA255 formed alkyl stacked with AEE. The TYR105 side chain formed a Pi-Pi stacked with AEE (Figure 10A). AEE and aspirin can form binding sites with the COX-1 protein as binding energies of −7.1 and −5.9 kJ, respectively (Table S4). Nine active site residues (GLY526, MET522, SER530, ALA527, ARG120, VAL116, LEU359, LEU357, MET113) were involved in AEE identification (Figure 10B). In protein COX-1, the SER530 side chains formed conventional hydrogen bonds with AEE. The ARG120 formed Pi-Cation stacked with AEE. The MET522 formed Pi-Sulfur stacked with AEE. VAL116, LEU359, LEU357, and MET113 formed alkyl stacked with AEE. ALA527 formed Pi-Sigma stacked with AEE. The GLY526 side chain formed an Amide-Pi stacked with AEE (Figure 10B).

## 3. Discussion

Inflammation is a protective response of the organism to clear external stimuli to maintain homeostasis during infection or injury [31]. Prolonged and uncontrolled inflammatory reactions can cause a variety of diseases, such as cardiovascular dysfunction, metabolic disorders, and autoimmune diseases [32]. Thrombosis and inflammation are closely related, inducing and enhancing each other’s biological effects [33,34]. Platelets can be directly involved in inflammatory reactions, which, in turn, leads to platelet activation [35], and AEE has been shown to have anti-inflammatory, thrombopreventive, and platelet-activating effects. This study investigated the effect of AEE on platelets of rats with carrageenan-induced thrombosis through platelet proteomics to clarify the regulatory network and target of platelet activation inhibition by AEE.

Carrageenan, as an inflammation inducer, can cause damage to vascular endothelial cells and activate the coagulation system, leading to thrombosis. It induces endothelial cells to release pro-coagulant materials and changes the permeability of the blood vessel wall and the fluidity of the blood, thereby creating conditions conducive to thrombosis [36,37]. Carrageenan has been widely used as a common drug for the modeling of thrombosis studies, such as the longshengzhi capsule, which reduces carrageenan-induced thrombosis by reducing the activation of platelets and endothelial cells [38]. The combination of Danhong injection plus tissue plasminogen activator ameliorates mouse tail thrombosis-induced by κ-carrageenan [39]. Antarctic krill oil from euphausia superba ameliorates carrageenan-induced thrombosis in a mouse model [40]. In this study, between the model and control groups, DEPs were mainly enriched in complement and coagulation cascades, innate immune response, acute-phase response, the positive regulation of B cell activation, inflammatory response, immune system process, zymogen activation, plasminogen activation, cellular response to cAMP, the common pathway of fibrin clot formation, intrinsic pathway of fibrin clot formation, platelet degranulation, and cell surface interactions at the vascular wall. This indicated that after the intraperitoneal injection of carrageenan in rats, carrageenan firstly destroys the vascular endothelial cells, causing endothelial cell damage, activates the complement system, and releases inflammatory mediators, which leads to an increase in vascular permeability, thereby leading to the entry of carrageenan into the circulatory system and the activation of platelets and their macrophages. The damaged endothelial cells adhere to the activated platelets and form thrombi. Activated platelets release large amounts of inflammatory mediators that support leukocyte chemotaxis, adhesion, and migration to sites of inflammation [41]. Platelets act as key immunomodulators of atherosclerosis progression by promoting monocyte migration to lesions and inducing a pro-inflammatory M1-like macrophage phenotype, leading to an increase in monocyte–platelet aggregates [42]. This shows that when endothelial cells are damaged, the site of damage recruits activated platelets and monocytes, resulting in severe thrombosis.

Platelet proteomics results showed that between the AEE and model groups, DEPs were mainly enriched in complement activation, ADP signaling through P2Y purinoceptor 12, cell surface interactions at the vascular wall, platelet activation, epoxide metabolic processes, platelet aggregation, the regulation of blood vessel remodeling, mTOR signaling, and the Ca2+ pathway. The pathways in this result were different from previous results, including mTOR signaling and ADP signaling at P2Y purinoceptor 12. MTOR is a serine/threonine protein kinase that exists in two distinct multi-protein complexes, mTOR complex 1 (mTORC1) and mTOR complex 2 (mTORC2) [43]. MTORC1 is sensitive to various signals, including nutrients, growth factors, and energy status, and mainly regulates cell growth, metabolism, and protein synthesis. MTORC2 mainly regulates the cell survival, proliferation, and organization of the cytoskeleton [44]. MTOR signaling could regulate platelet function. MTORC1 could promote the synthesis of proteins in platelets, which may be crucial for platelet activation and aggregation-related protein production [45,46]. For example, some membrane proteins involved in platelet adhesion and aggregation may increase their expression through the mTORC1-regulated synthetic pathway, thereby enhancing platelet aggregation and promoting thrombosis [47]. In addition, mTOR signaling may affect signal transduction pathways within platelets that regulate platelet responsiveness to stimuli [48]. TXA2 is a prostaglandin released by platelet activation, which causes platelets to aggregate and form thrombi and is an important physiological aggregation factor. Cyclooxygenase inhibitors block the synthesis of TXA2 precursors by acting on COX-1, thereby inhibiting the production of TXA2 and platelet aggregation. Aspirin blocks TXA2 production by inactivating COX-1 through the acetylation of the hydroxyl group on the serine residue at position 530 of the COX-1 polypeptide chain. AEE can also inhibit TXA2 production [26]. Therefore, this study predicted the binding of AEE and aspirin to COX-1 protein via the molecular docking method and found that the binding of AEE was higher than that of aspirin, indicating that AEE may have a stronger effect on COX-1 than aspirin. P2Y12 is an ADP receptor existing on the surface of platelets. After platelet activation, a high concentration of ADP is released to bind to this receptor and activate integrin αIIbβ3 to cause platelet aggregation, and this receptor is also involved in TXA2 production and the amplification of the release of various granules in platelets, so the P2Y12 receptor plays an essential role in the process of thrombosis [49]. Ticagrelor and cangrelor, representative of the cyclopentyltriazolepyrimidines, are present in the form of active drugs that bind directly and reversibly to the P2Y12 receptor [50]. This study predicted the binding of AEE, the ticagrelor, and cangrelor to the P2Y12 protein via the molecular docking method and found that the binding of AEE was similar to that of the P2Y12 inhibitor, indicating that AEE could act through the P2Y12 protein to inhibit platelet activation.

Metabolomics of heart and abdominal aortic tissues showed that differential metabolites were mainly involved in nicotinate and nicotinamide metabolism, glycerophospholipid metabolism, steroid biosynthesis, the citric acid cycle, phenylalanine metabolism, phenylalanine, tyrosine, and tryptophan biosynthesis, and glutathione metabolism. The thromboprophylaxis of AEE was associated with the metabolism of amino acids, an improvement in lipid metabolism, a reduction in oxidative stress, and the repair of energy metabolism damage. The results of this study are similar to previous results, which indicates that the effect of AEE on thrombotic rats has an effect of the whole organism.

## 4. Materials and Methods

### 4.1. Chemicals

AEE was prepared by the Lanzhou Institute of Husbandry and Pharmaceutical Sciences of CAAS (Lanzhou, China). Carboxymethylcellulose sodium (CMC-Na) was supplied by the Tianjin Chemical Reagent Company (Tianjin, China). The rat platelet isolate kit (P8570) was supplied by Solarbio (Beijing, China). MS-grade formic acid was purchased from TCI (Shanghai, China), and κ-carrageenan was provided by Sigma-Aldrich (St. Louis, MO, USA). Acetonitrile (MS grade) and methanol (MS grade) were purchased from Thermo Fisher Scientific Corporation (Waltham, MA, USA). The other reagents with analytical grade were purchased from the Sinopharm Group (Shanghai, China). AEE was dissolved in a 0.5% sodium CMC-Na solution to form a storage suspension; then, the rats were given the corresponding amount of the drug according to their body weight.

### 4.2. Animal Experiment and Study Design

All experimental protocols and procedures were approved by the Institutional Animal Care and Use Committee of Lanzhou Institute of Husbandry and Pharmaceutical Science of the Chinese Academy of Agricultural Sciences (Approval Date: 26 April 2023). Animal welfare and experimental procedures were performed strictly by the Guidelines for the Care and Use of Laboratory Animals issued by the US National Institutes of Health. Twenty-four Wistar male rats (9 weeks old) weighing 200~220 g were purchased from the Lanzhou Veterinary Research Institute, Chinese Academy of Agricultural Sciences (Lanzhou, China). Rats were housed in animal rooms under standard conditions (12 h light/dark cycle, humidity of 48 ± 10%, and temperature of 20 ± 2 °C) and were provided with food and water ad libitum. The rats were acclimatized for one week before the start of the study. The rats were randomly divided into three groups (*n* = 8), such as the control, model, and AEE group (AEE, 18 mg∙kg^−1^∙d^−1^ b.w.). Briefly, Wistar rats were given either the vehicle alone (0.5% CMC-Na) or a vehicle combination containing AEE (18 mg∙kg^−1^∙d^−1^ b.w.) for 7 d. Then, all groups of rats, except those in the control group, were injected with 20 mg/kg κ-carrageenan to induce thrombosis. After 24 h, all the rats were anesthetized by an intraperitoneal injection of sodium pentobarbital (80 mg∙kg^−1^ b.w.), and then the blood was collected through the abdominal aorta, and platelets were isolated.

### 4.3. Proteomics Analysis

#### 4.3.1. Platelet Collection and Preparation

After treating rats, platelets were collected according to the method of the rat platelet isolate kit. Firstly, red blood cells were removed by sedimentation, and then platelet-rich plasma was obtained by centrifugation at 250~350× *g* with a horizontal rotor for 15 min at room temperature. The PRP was collected, added to the washing solution, and centrifuged at 500× *g* for 20 min to obtain platelet precipitate. The collected samples were frozen with liquid nitrogen and stored in the refrigerator at −80 °C for subsequent experiments. Platelet protein preprocessing methods are described in the Supplementary Materials.

#### 4.3.2. Chromatography Conditions

Nanoflow reversed-phase chromatography was performed on a nanoElute liquid chromatography system (Bruker Daltonics, Bremen, Germany). Specific parameters are provided in the Supplementary Materials.

#### 4.3.3. Mass Spectrometry Conditions

Liquid chromatography was coupled online to a hybrid TIMS quadrupole TOF mass spectrometer (Bruker timsTOF Pro, Bremen, Germany) via a CaptiveSpray nano-electrospray ion source. Specific parameters are provided in the Supplementary Materials.

#### 4.3.4. Protein Identification and Bioinformatics Analysis

Protein identification-specific parameters are provided in the Supplementary Materials. Compared with the model, the proteins with a *p*-value less than 0.05 and higher than 2-fold changes (or lower than 0.5) were considered as DEPs for further analysis. Gene Ontology (GO) annotations of the proteins were performed using the Blast2GO program (version 3.3.5) (http://www.geneontology.org, accessed 16 April 2024). The corresponding KEGG pathways of the proteins were also extracted and mapped in the KEGG database (https://www.genome.jp/kegg/, accessed 16 April 2024). GO enrichment and KEGG pathway enrichment analysis were applied based on Fisher’ exact test. Functional protein–protein interaction (PPI) networks were analyzed using STRING (http://string-db.org/, accessed 16 April 2024).

### 4.4. Metabolomics Analysis

#### 4.4.1. Rat Heart and Abdominal Aorta Sample Preparation

Samples were processed as previously described [29]; a 100 mg heart sample was added to 1 mL methanol/water (4:1, *v*:*v*) for homogenate, followed by 1 min of vortex mixing and 8 min of ultrasonic extraction. After standing (10 min on ice) and centrifugation (14,000× *g*, 10 min, 4 °C), 0.8 mL of the supernatant was transformed and evaporated to dry. The residue was redissolved in 200 μL methanol/water (4:1, *v*:*v*) for metabolomics analysis. The supernatant was filtered through the 0.22 μm filter membrane and subsequently analyzed by UPLC-QTOF-MS/MS. Abdominal aortic samples were manipulated, as described above.

#### 4.4.2. UPLC-QTOF-MS/MS Conditions

UPLC-QTOF-MS/MS conditions were the same as those previously described. Briefly, chromatographic analysis was carried out using the 1290 UPLC system (Agilent Technologies, Santa Clara, CA, USA). Protein identification-specific parameters are provided in the Supplementary Materials.

#### 4.4.3. Metabolomics Data Analysis

Metabolites identified specific parameters, which are provided in the Supplementary Materials. Finally, the metabolites with VIP > 1 and *p* < 0.05 were screened for metabolite variability analysis in different groups. Then, information on the differential metabolites of concern was finalized by performing the spectral library comparison in the Human Metabolome Database (HMDB) (http://www.hmdb.ca/, accessed 9 June 2024). According to the information on differential metabolites, MetaboAnalyst 6.0 (http://www.metaboanalyst.ca/, accessed 19 June 2024) was used for the analysis of relevant metabolic pathways.

### 4.5. Molecular Docking

The RCSB PDB database (http://www.rcsb.org/, accessed 12 July 2024) was used to download the crystal structures of the relevant proteins and save them in PDB format. Swiss-PdbViewer 4.1 was used to patch the residues. AutoDock Tools 1.5.7 was used to add water and hydrogen atoms to protein. Molecular docking was performed using the AutoDock Vina software(version 1.2.0) and visualized using Discovery Studio software (version 4.0). In this study, AEE, ticagrelor, cangrelor, and P2Y12 protein (PDB: 4NTJ), COX-1 protein (PDB: 6Y3C) binding modes were explored, and the docking results between different drugs and key targets were evaluated based on the obtained binding energy values.

## 5. Conclusions

The thromboprophylaxis of AEE was associated with the metabolism of amino acids, an improvement in lipid metabolism, a reduction in oxidative stress, and the repair of energy metabolism damage. AEE regulated the expression of several proteins in rat platelets in the κ-carrageenan-induced rat tail thrombosis model. A total of 348 DEPs were identified, of which 87 were up- and 261 were down-regulated. AEE was able to reverse the changes induced by κ-carrageenan to some extent. DEPs were enriched to such two novel pathways as mTOR signaling and ADP signaling at P2Y purinoceptor 12. AEE had a better binding force to both the COX-1 protein and P2Y12 protein. These findings provide a novel theoretical basis for the inhibitory effect of AEE on platelet activation.

## Data Availability

The data that support the findings of this study are available from the corresponding author upon reasonable request. Some data may not be made available because of privacy or ethical restrictions. The mass spectrometry proteomic data were deposited to the ProteomeXchange Consortium (https://proteomecentral.proteomexchange.org, accessed on 2 October 2024) via the iProX partner repository [51,52] with the dataset identifier PXD054906.

## Supplementary Materials

The following supporting information can be downloaded at: https://www.mdpi.com/article/10.3390/ijms251910747/s1.

## Abbreviations

AEE: aspirin eugenol ester; CMC-Na: carboxymethylcellulose; DEPs: differentially expressed proteins.
