# Supplementary material for: Platelet Proteomics and Tissue Metabolomics Investigation for the Mechanism of Aspirin Eugenol Ester on Preventive Thrombosis Mechanism in a Rat Thrombosis Model
Source: Int J Mol Sci. 2024 Oct 6;25(19):10747. doi: 10.3390/ijms251910747 (PMC11476519; doi:10.3390/ijms251910747)
Supplement: Supplementary file 1 [file ijms-25-10747-s001.zip › Supplementary Materials Figures and Tables.pdf]

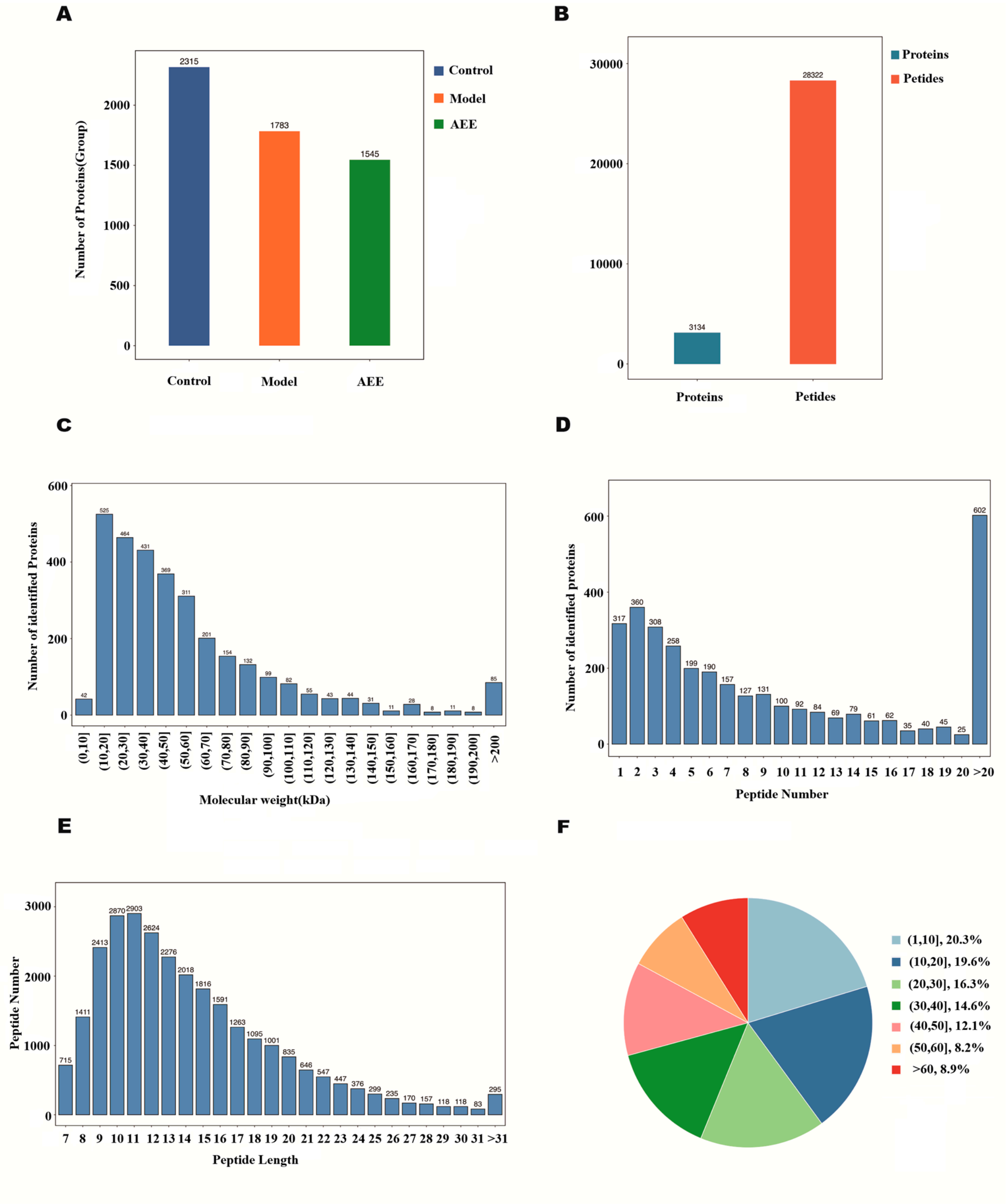

Fig. S1 Identification and quantitative evaluation of proteins. (A) Number of proteins. (B) Identified peptides and proteins. (C) Protein molecular weight distribution. (D) Peptide quantity distribution. (E) Peptide length distribution. (F) Distribution of peptide sequence coverages.

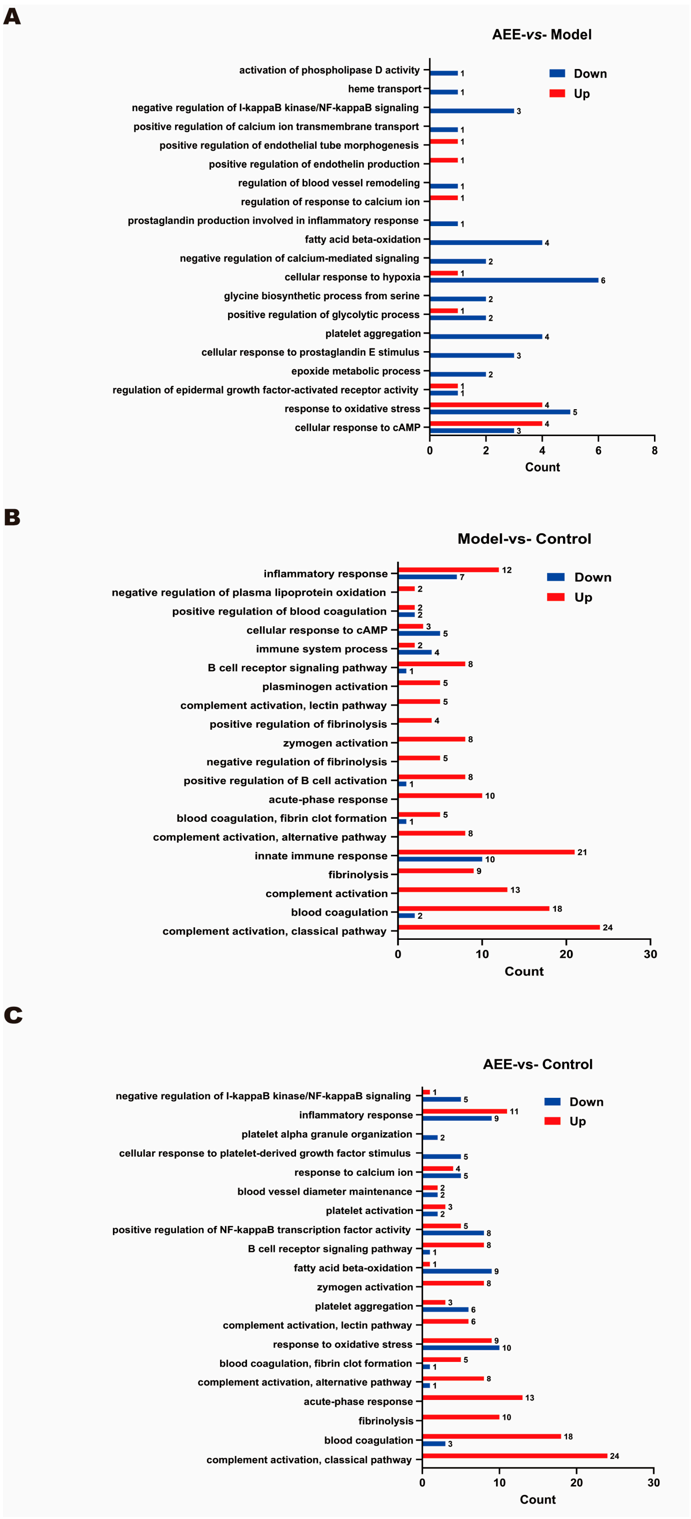

Fig. S2 GO enrichment analysis of DEPs, up and down-regulated proteins. (A) AEE group *vs.* Model group. (B) Model group *vs.* Control group. (C) AEE group *vs.* Control group.

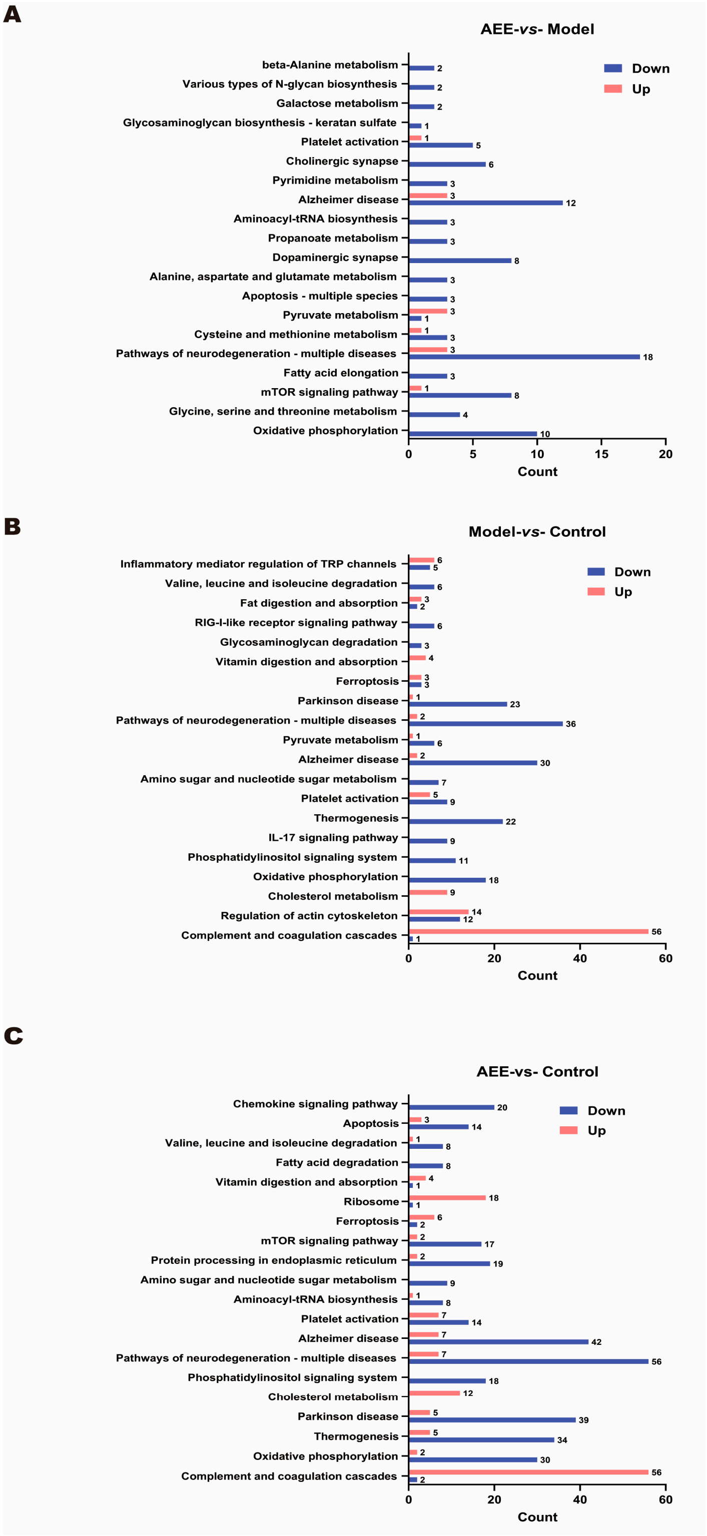

Fig. S3 KEGG pathway analysis of DEPs, up and down-regulated proteins. (A) AEE group *vs.* Model group. (B) Model group *vs.* Control group. (C) AEE group *vs.* Control group.

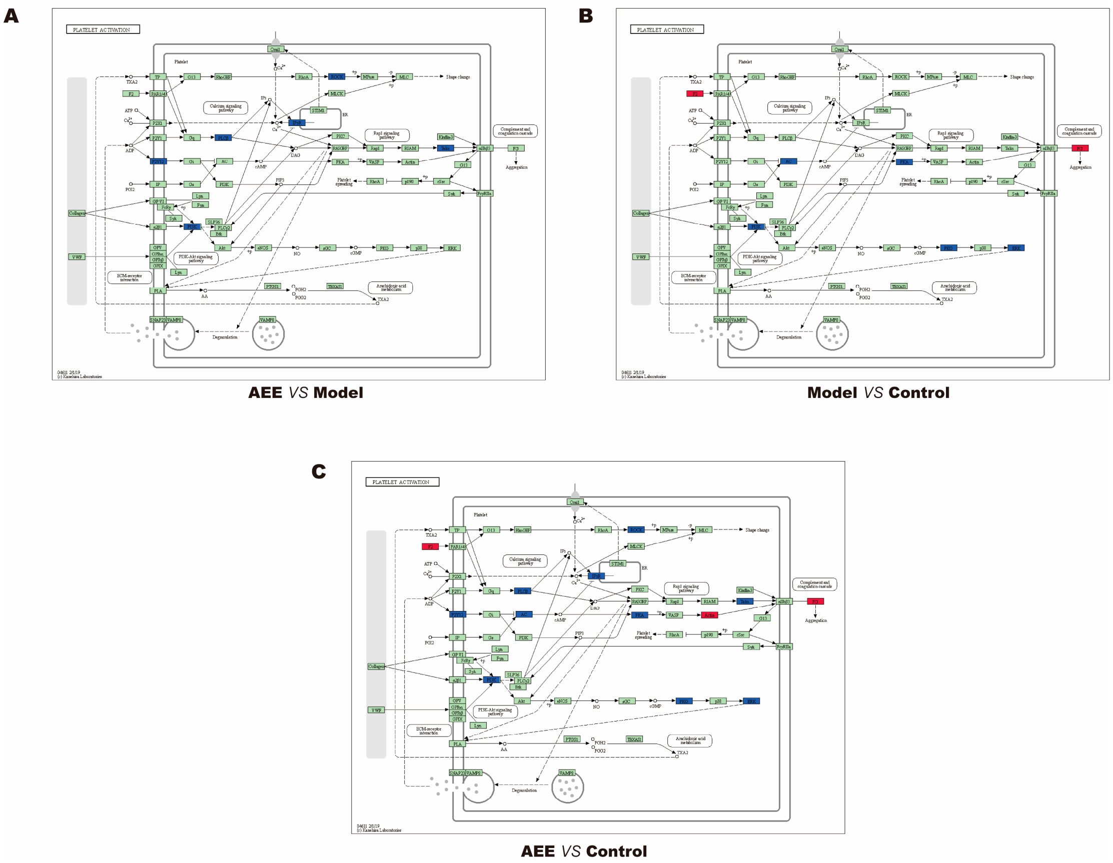

Fig. S4 KEGG map analysis of DEPs. (A) AEE group *vs.* Model group. (B) Model group *vs.* Control group. (C) AEE group *vs.* Control group.

Table S1 Sample absorbance and concentration

Number	Absorbance1	Absorbance2	Absorbance3	Average absorbance	Concentration （ug/uL）
Control 1	0.364	0.323	0.316	0.334	3.099
Control 2	0.249	0.252	0.251	0.251	2.315
Control 3	0.182	0.180	0.202	0.188	1.729
Control 4	0.257	0.234	0.224	0.238	2.200
Control 5	0.446	0.454	0.431	0.444	4.122
Model 1	0.346	0.330	0.333	0.336	3.117
Model 2	0.195	0.165	0.163	0.174	1.601
Model 3	0.248	0.245	0.259	0.251	2.315
Model 4	0.113	0.112	0.110	0.112	1.014
Model 5	0.131	0.129	0.138	0.133	1.211
AEE 1	0.199	0.195	0.189	0.194	1.788
AEE 2	0.106	0.111	0.105	0.107	0.974
AEE 3	0.172	0.171	0.172	0.172	1.576
AEE 4	0.130	0.117	0.111	0.119	1.086
AEE 5	0.098	0.100	0.101	0.100	0.902

Table S2 statistics of differential metabolites in heart of rats.

No	RT	VIP	Formula	Metabolites	SM	m/z	Fold Change AEE/Model
1	3.034	1.08747	$C_6H_6O_2$	Hydroquinone	ESI+	110.03483	2.49473*
2	3.74	1.19552	$C_5H_7N_5O$	FAPy-adenine	ESI+	153.03984	3.23817*
3	5.356	1.1928	$C_7H_9N_2O$	1-Methylnicotinamide	ESI+	137.04474	2.75365*
4	8.076	1.09282	$C_5H_{15}NO_4P$	Phosphorylcholine	ESI+	184.05873	3.40597*
5	16.336	1.3069	$C_8H_{15}NOS_2$	Lipoamide	ESI+	205.3410	5.84363*
6	16.459	1.04902	$C_5H_{15}NO_4P$	Phosphorylcholine	ESI+	184.1507	1.69416
7	21.337	1.07495	$C_{27}H_{44}O$	7-Dehydrocholesterol	ESI+	384.24915	2.56352*
8	5.81	1.41058	$C_{10}H_{12}O_2$	2-Phenylbutyric acid	ESI-	164.0715	3.00246*

RT, retention time; VIP, variable importance in the projection; SM, scan mode; +, metabolites identified in positive mode; -, metabolites identified in negative mode. Metabolites identified in both positive and negative modes; *$P < 0.05$.

Table S3 statistics of differential metabolites in abdominal aorta of rats.

No	RT	VIP	Formula	Metabolites	SM	m/z	Fold Change AEE/Model
1	3.177	1.0012	$C_{10}H_{17}N_3O_6S$	Glutathione	ESI+	307.08075	0.373996*
2	5.073	1.16917	$C_5H_5N_5$	Adenine	ESI+	135.1009	5.18844*
3	5.314	1.10117	$C_7H_9N_2O$	1-Methylnicotinamide	ESI+	137.0449	7.73158*
4	5.878	1.1285	C_8H_8O	Phenylacetaldehyde	ESI+	120.08005	1.8395*
5	5.915	1.10058	$C_9H_{10}O_3$	Desaminotyrosine	ESI+	166.0846	0.521925
6	6.543	1.03319	$C_6H_{10}O_4$	2-Methylglutaric acid	ESI+	146.1165	3.64745*
7	6.874	1.07917	$C_{11}H_{11}NO_3$	Indolelactic acid	ESI+	205.0953	1.75641
8	10.985	1.22974	$C_{19}H_{30}O_2$	Etiocholanolone	ESI+	290.2673	3.54425*
9	11.555	1.17914	$C_9H_{11}NO_2$	Phenylalanine	ESI+	165.09	14.0043*
10	5.854	1.19868	$C_{10}H_{12}O_2$	3-Phenylbutyric acid	ESI-	164.0696	4.28269*

RT, retention time; VIP, variable importance in the projection; SM, scan mode; +, metabolites identified in positive mode; -, metabolites identified in negative mode. Metabolites identified in both positive and negative modes; *$P < 0.05$.

Table S4 Docking results of compounds and target proteins

Target	Compound	Energy (kcal·mol^{-1})
P2Y12	AEE	-8.2
	Ticagrelor	-9.2
	Cangrelor	-6.9
COX-1	AEE	-7.1
	Aspirin	-5.9
