# Supplementary material for: Platelet Proteomics and Tissue Metabolomics Investigation for the Mechanism of Aspirin Eugenol Ester on Preventive Thrombosis Mechanism in a Rat Thrombosis Model
Source: Int J Mol Sci. 2024 Oct 6;25(19):10747. doi: 10.3390/ijms251910747 (PMC11476519; doi:10.3390/ijms251910747)
Supplement: Supplementary file 1 [file ijms-25-10747-s001.zip › Supplementary Materials.pdf]

1. Platelet protein extraction

Rat platelet samples were transferred into low protein binding tubes and lysed with 300 μL lysis buffer supplemented with 1mM PMSF. Then samples were further lysed with sonication. The parameters were set as 1s /1s intervals and 80 W for 2 min. After sonication, the samples were centrifuged at 12000*g* for 10 min at 4°C to remove insoluble particles, repeat once to further exclude precipitation. The protein concentration was determined by BCA assay. The protein samples were aliquoted to store at -80°C.

2. Trypsin digestion

According to the measured protein concentration (Table S1), the same quality protein was taken from each sample, and different groups of samples were diluted to the same concentration and volume. By adding 25 mM DTT of corresponding volume into the above protein solution to make the DTT final concentration about 5mM, and incubate at 55°C for 30 min. Then the corresponding volume of iodoacetamide was added so that the final concentration was about 10mM, and placed in the dark for 15 min at room temperature. Then 6 times volume of precooled acetone in the above system to precipitate the protein, and place it at - 20 °C for more than four hours or overnight. Subsequently, the sample was centrifuged at 8000g for 10 min at 4 °C for collecting the precipitate. According to the amount of protein, the corresponding volume of

enzymolysis diluent (protein: enzyme 50:1, 100 μg of protein add 2 μg of enzyme) was added to redissolve the protein precipitate, then the solutions were incubated for digestion at 37°C for 12 h. Finally, samples were lyophilized or evaporated after enzymolysis and stored at -80°C.

3. Peptide segment desalination

The digested peptides were desalted by SOLA™ SPE 96-plate Column. Firstly, the column was activated with 200 μL of methanol and repeated 2 times for a total of 3 times. Then 200 μL of equilibrium solution (0.1% formic acid, 100% water) to activate the column, repeat 2 times. The samples were loaded on the column twice. Then the column was washed with 0.1% formic acid aqueous solution 3 times. Finally, the peptides were eluted with 150 μL of 50% acetonitrile-water (containing 0.1% formic acid) 3 times and were lyophilized.

4. Proteomics Analysis

4.1 Chromatography Conditions

Peptides were separated in 90 min at a flow rate of 300 nL/min on a 25 cm × 75 μm column (1.6 μm C18, ionopticks). Mobile phases A and B were 0.1% formic acid solution and ACN with 0.1% formic acid, respectively. The total run was 90 min (0~75 min，2-22% B；75~80 min，22-37% B；80~85 min，37-80% B；85~90min，80% B).

4.2 Mass Spectrometry Conditions

Capillary voltage was 1.4 kV, dry gas temperature was 180°C, and

dry gas flow rate was 3.0 L/min. The dual TIMS analyzer was operated at a fixed duty cycle close to 100% using equal accumulation and ramp times of 100 ms. We performed DDA in PASEF mode with 10 PASEF scans per topN acquisition cycle. The full MS scan range was set from 100 to 1700 m/z. The ion mobility range was 0.6-1.6 vs/cm^2, and the collision energy range was 20-59 ev.

5. Protein identification specific parameters

The LC-MS/MS raw data were imported in Maxquant (Version1.6.17.0) for labeling free quantification analysis and the search engine was Andromeda. The main parameters were set as followed: enzyme was set as trypsin, max missed cleavages was 2, peptide mass tolerance was set at ±20 ppm, fragment mass tolerance was 0.5 Da， and fixed modification was carbamidomethyl. To minimize the false positive results, the cutoff of FDR less than 0.01 was applied in the protein identification.

6. Metabolomics Analysis

6.1 UPLC-QTOF-MS/MS Conditions

The mobile phase consisted of a linear gradient system of (A) water with 0.1% formic acid and (B) acetonitrile with 0.1% formic acid, 0-2 min, 98% A; 2-11 min, 98-55% A; 11-15 min, 55-30% A; 15-22 min, 30-2% A; 22-27 min, 2% A. High-definition mass spectrometer Agilent 6530 Q-TOF (Agilent Technologies, USA) was used to perform the mass data

acquisition in positive or negative electrospray ionization source (ESI+ or ESI-). The optimal conditions of MS analysis were as followed: MS data was collected in centroid mode from 50-1000 m/z, scan rate was 1 spectra/second, desolvation gas rate was 10 L/min, gas temperature was 350°C, the nebulizer pressure was 45 psig, fragment voltage was 135 V, skimmer voltage was 65 V, and the capillary voltages were 4.0 KV in positive mode and 3.5 KV in negative mode, respectively.

6.2 Metabolomics Data Analysis

The original data were processed using MS-DIAL V 4.38, the two-dimensional data matrix including mass-to-charge ratio, peak area and retention time were obtained. The obtained data were subjected to multivariate statistical analysis using SIMCA-P (version 13.0, Umetrics AB, Umea, Sweden) software, and the results were presented as principal component analysis (PCA) score plots and partial least squares discriminant analysis (OPLS-DA) score plots, respectively.
